# Seroprevalence and Clinical Features of Scrub Typhus among Febrile Patients Attending a Referral Hospital in Kathmandu, Nepal

**DOI:** 10.3390/tropicalmed6020078

**Published:** 2021-05-13

**Authors:** Anil Pokhrel, Binod Rayamajhee, Saroj Khadka, Sandeep Thapa, Samjhana Kapali, Sher Bahadur Pun, Megha Raj Banjara, Prakash Joshi, Binod Lekhak, Komal Raj Rijal

**Affiliations:** 1 Central Department of Microbiology, Tribhuvan University, Kirtipur 44618, Nepal; pokhrel.anil1989@gmail.com (A.P.); khadkasaroj09@gmail.com (S.K.); samjhanakapali123@gmail.com (S.K.); banjaramr@gmail.com (M.R.B.); binodlekhak9@gmail.com (B.L.); 2 Department of Infection and Immunology, Kathmandu Research Institute for Biological Sciences (KRIBS), Kathmandu 44700, Nepal; rayamajheebinod@gmail.com; 3 Kathmandu Centre for Genomic Research Laboratory (KCGRL), Kathmandu 44700, Nepal; biotechlbef@gmail.com; 4 Sukraraj Tropical and Infectious Disease Hospital, Kathmandu 44600, Nepal; drsherbdr@yahoo.com; 5 Kanti Children’s Hospital, Kathmandu 44600, Nepal; dr.prakash346088@gmail.com

**Keywords:** scrub typhus, *Orientia tsutsugamushi*, fever, seroprevalence, Nepal

## Abstract

(1) Background: Scrub typhus (ST) is endemic to Nepal. It is often underdiagnosed and misdiagnosed due to non-specific clinical presentation coupled with limited microbiological facilities, leading to adverse clinical outcomes. This study aimed to assess the seroprevalence of scrub typhus in febrile patients attending Sukraraj Tropical and Infectious Disease Hospital (STIDH), Nepal, from August 2018 to April 2019. (2) Materials and Method: Blood/serum samples and clinical and demographic data of adult febrile patients (≥19 years) who attended or were referred to the hospital were collected after obtaining written informed consent from the participants excluding immunocompromised individuals. Collected blood/serum samples were subjected to hematological, biochemical, and serological tests. A serological test for scrub typhus was performed using the ImmuneMed scrub typhus rapid diagnostic test kit. Data generated were analyzed using SPSS software version 24.0. (3) Results: Amongst the 2070 febrile patients, 462 (22.3%) were seropositive to at least one etiological agent of febrile illnesses (scrub typhus: 253 cases, dengue: 101 cases, leptospirosis: 9, brucellosis: 52, malaria: 9 and kala-azar: 20 cases). Scrub typhus accounted for 12.2% (n = 253) of total febrile illnesses followed by dengue (4.9%, n = 101). Mixed seropositivity of scrub typhus with dengue, brucellosis, and typhoid was found in 12 (0.6%), 9 (0.4%), and 5 (0.2%) cases, respectively. Among 253 scrub typhus patients, 53.4% were female. Among the 154 patients, the most common symptoms were fever (100%), headache (79.2%), sweating (70.1%), breathing difficulty (51.3%), redness of the eye (43.5%), and pathognomonic eschar was observed in 9.1% patients. Fifty percent of scrub typhus patients had low platelet count and >30% of patients had an elevated level of liver enzymes (such as serum glutamic oxaloacetic transaminase (SGPT) and serum glutamic pyruvic transaminase (SGOT). (4) Conclusion: Scrub typhus is a considerable cause of febrile illness in Nepal. Females apparently have a higher chance of acquiring scrub typhus. ST presents nonspecific clinical presentation. The diagnostic dilemma of typhus patients can be minimized by the early monitoring of ST-associated symptoms. The country’s health system needs to be strengthened for early outbreak detection, and immediate response actions against scrub typhus to control the future outbreak of ST.

## 1. Introduction

Mite-borne scrub typhus (ST), also known as tsutsugamushi disease, is an acute febrile illness caused by the obligatory intracellular bacteria *Orientia tsutsugamushi*. It is transmitted through the bite of the infected larval form (chigger) of *Leptotrombidium* mites [1,2,3,4,5]. Even though ST is considered endemic in the so-called ‘tsutsugamushi triangle’ of the Asia-Pacific region including Nepal, it has emerged in countries outside of the traditional ‘tsutsugamushi triangle’ such as in Chile, UAE, Cameroon, Kenya, Congo, Djibouti, and Tanzania [5,6]. The disease affects >1 million patients annually in the endemic area, and one billion people of all age groups are at risk of infection (3,7). After an incubation period that ranges from 6 to 21 days (usually 10–12 days), ST presents with ‘flu-like’ symptoms and is characterized by fever, rash, headache, eschar, pneumonitis, leading to severe systemic multi-organ failure including renal failure, respiratory dysfunction and involvement of central nervous system (CNS) in untreated cases [1,4,7]. The mortality among untreated patients is ~6%, with a wide range of 0–70% depending on the endemic area and the patient’s immune status [5].

The infectious cause of ST and other febrile illnesses remains poorly characterized in low- and middle-income countries (LMICs) due to non-specific clinical presentation, limited awareness, low index of suspicion among clinicians, and poor diagnostic facilities [1,8,9,10]. In Nepal, *Salmonella enterica* serovars Typhi and Paratyphi A are regarded as the leading cause of febrile illness [11], while ST is responsible for about 36% of undifferentiated febrile illnesses (UFI) [12]. Although the history of ST in Nepal dates back to 1981 [13], a surge in scrub typhus cases was recorded in Nepal after the 2015 earthquake [14]. Although scrub typhus is endemic in Nepal, there is no established clinical marker to distinguish typhus from enteric fever and the classic signs and symptoms of rickettsial disease (headache, fever, rash, and eschar) are rarely detected in Nepali patients [10,15]. Many cases of murine typhus, scrub typhus, and leptospirosis is often diagnosed as enteric fever [10]. About 50% of ST cases are clinically diagnosed as enteric fever in Nepal where the use of the Widal test is widespread [10]. A possible cross-reactivity between typhoid, typhus, and leptospirosis with a Widal test might lead to misdiagnosis of typhoid fever particularly in typhoid endemic regions such as in Nepal where low background typhoid antibodies are present in the normal population [8] eventually leading to possible misdiagnosis of ST. There is a possibility of cross-reaction between the serological test for ST and leptospirosis [16] leading to misdiagnosis. Diagnosis of typhus is based on a four-fold increase in serological titers, which is not usually obtained until 3 weeks post-infection, can be too late for clinical management. Furthermore, polymerase chain reaction (PCR) tests are less sensitive, expensive, and often not readily available in resource-limited countries like Nepal [4,8], and indirect immunofluorescence assay (IFA)-the gold standard technique to diagnose ST, is relatively expensive and usually not performed in the clinical setting of Nepal. In essence, studies have reported an excellent response to treatment, so timely antimicrobial therapy may help to prevent complications associated with ST [2,4,17]. Despite the increased incidence of ST in Nepal, so far there is no clear epidemiological picture. This study aimed to assess seroprevalence and associated clinical symptoms of scrub typhus and mixed seropositivity among febrile patients attending a referral hospital in central Nepal. 

## 2. Material and Methods

### 2.1. Study Design and Sample Size

A hospital-based prospective cross-sectional study was carried out from August 2018 to April 2019. A total of 2070 blood samples were collected from the following participants: adult febrile patients (≥19 years, axillary temperature >38 °C) attending the outpatient clinic and/or admitted in Sukraraj Tropical and Infectious Disease Hospital (STIDH), and adult febrile patients clinically examined at a different hospital but referred to STIDH for laboratory diagnosis only. Patients with a known immunocompromised condition were excluded from the study.

### 2.2. Sample and Clinical Data Collection 

Following the written informed consent, ~10 mL of venous blood was collected by trained laboratory staff, and then the blood was divided into three aliquots for serological, biochemical, and hematological tests. Blood specimens were collected in a plain tube, gel tube, and Ethylenediamine tetra acetic acid EDTA tube for serological, biochemical, and hematological investigations, respectively. Blood for serological and biochemical investigations was allowed to clot and centrifuged at 5000 rpm for 5 min to separate the serum. The serum specimen was then stored at −20 °C until further processing. Clinical and demographic characteristics including travel history were collected by using a structured questionnaire. Physical examination findings such as organomegaly were recorded under the supervision of a physician of the STIDH.

### 2.3. Sample Processing

The serum samples of the patients were subjected to biochemical, hematological, and serological analysis. 

### 2.4. Biochemical Test

Separated serum was used to investigate the liver enzymes (alkaline phosphatase, serum glutamic pyruvic transaminase (SGPT), serum glutamic oxaloacetic transaminase (SGOT)), bilirubin, and creatinine level. The investigation was performed by using an automated system (Erba XL 200, Erba. Mannheim, UK). The reference range for different hematological and biochemical parameters was used as suggested by the hospital guidelines (Supplementary Materials).

### 2.5. Hematological Test

White Blood Corpuscle (WBC) counting was performed as total leucocytes count (TLC), i.e., total leucocytes present per mm^3^ of blood. WBC count was performed by Sysmex XL 330 automated analyzer (Sysmex, Singapore).

### 2.6. Preparation of Thin Smear 

For the detection of the malarial parasite, one drop of fresh blood was placed on one end of a clean and dry glass slide and spread on the opposite end using another clean slide held at an angle of about 30°. Smear was allowed to air dry, placed over the staining rack, and fixed by using 2 drops of absolute methanol [18].

### 2.7. Serological Test

Dengue, leptospirosis, scrub typhus, and kala-azar were tested using rapid diagnostic test (RDT) kits as per the manufacturer’s instructions. Enteric fever was screened using the Widal test and brucellosis was tested by slide agglutination test. Malaria was first screened by RDT and confirmed by thin and thick smears. The results were validated by proficient laboratory staff of STIDH. The list of test kits used for the diagnosis of different diseases in this study is presented in Table 1.

ImmuneMed Scrub typhus rapid test kit is an immunochromatographic diagnostic test kit against scrub typhus that can diagnose the disease progression and reinfection status since IgG and IgM are analyzed [19], furthermore, various serotypes can be detected using this kit [19]. Blood specimen were considered seropositive to ST if both or either of IgG or IgM was detected.

### 2.8. Data Analysis

Data obtained during the study were maintained daily in Microsoft Excel 2016 before statistical analysis and it was later exported to the SPSS for Windows Version 24.0 for further statistical analysis. Frequency distribution and the correlation between different variables (χ^2^-test) were analyzed and calculated using SPSS. A *p*-value of <0.05 was considered statistically significant. Figures and graphs were created using OriginLab 2018 and QGIS 3.10.8.

### 2.9. Ethical Statement

This study protocol was reviewed and approved by the Nepal Health Research Council (NHRC), Nepal (Reg. No. 522/2018). Written informed consent was taken prior to the collection of specimens from each study participant.

## 3. Results

### 3.1. Demographic and Geographic Distribution of Patients 

**Figure 1:**
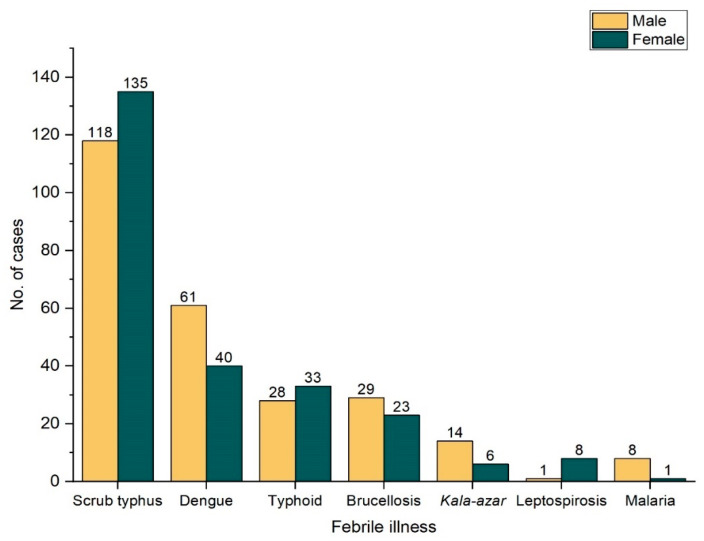
Distribution of Number of cases in different febrile illness.

In this study, males constituted a higher number of patients (57.5 %, n = 1191) where the age of patients ranged from 19 to 96 years with the mean age being 38.13 ± 16.61 years. The highest number of patients were from the age group 20–39 years (Table 2a). The gender-wise distribution of patients with different recorded febrile illnesses is depicted in Figure 1. 

**Figure 2:**
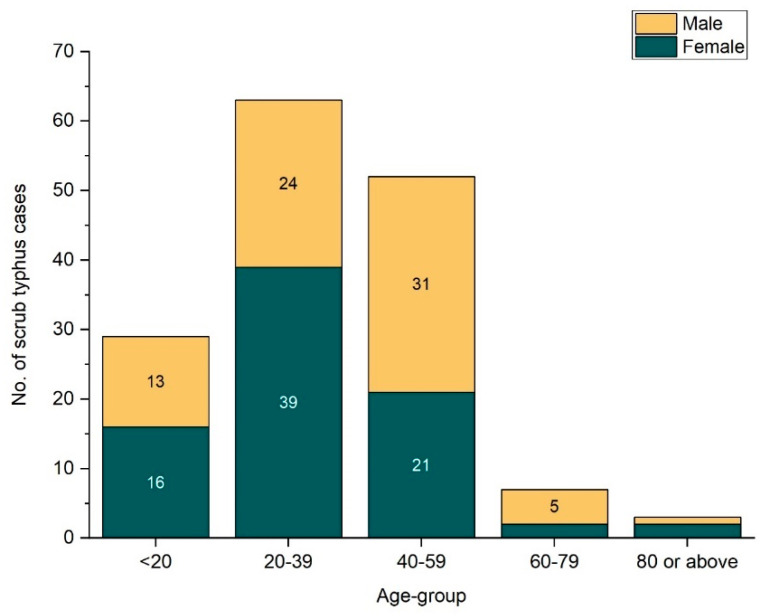
Age and sex wise distribution of scrub typhus cases.

The mean age of 154 ST patients was 36.42 ± 15.6 years with the age range being 19 years through 82 years. Among 253 ST patients, 53.4% were female, however, among 154 scrub typhus patients, 51.9% (n = 80) were female (Figure 2).

### 3.2. Biochemical and Hematological Investigations

Biochemical and hematological features were available for only 139 out of 154 ST patients. WBC count was low in 11 (7.9%) patients, decreased platelet count was observed in 77 (55.4%) patients (Table 2C), and thrombocytopenia was significantly associated with ST (*p*-value <0.01). Alkaline phosphatase, serum glutamic pyruvic transaminase (SGPT), and serum glutamic oxaloacetic transaminase (SGOT) levels were found to be higher than the normal range in 44 (31.7%), 53 (38.1%), and 85 (61.2%) ST patients, respectively. There was no significant association between the serum concentration of alkaline phosphatase and SGOT with ST. Raised SGPT, on the other hand, was significantly associated with ST (*p*-value < 0.01) The levels of total bilirubin (BT), direct bilirubin (BD), and serum creatinine were high in 9 (6.5%), 14 (9.4%), and 13 (10.1%) ST patients, respectively (Table 2C). 

**Figure 3:**
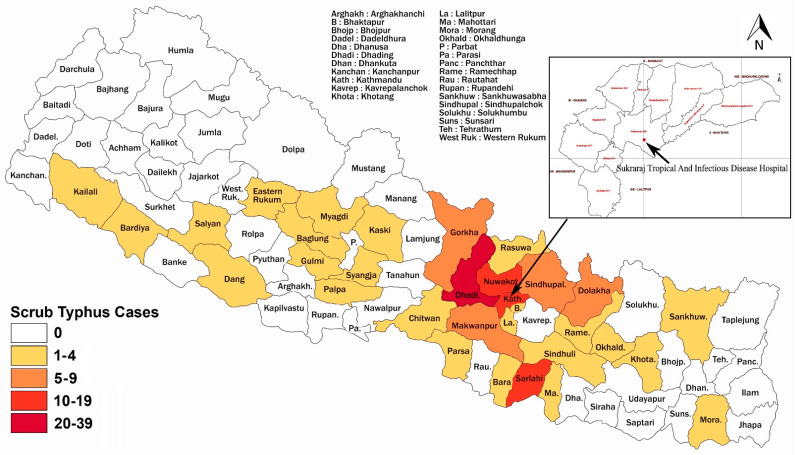
Geographical distribution of ST patients recorded in this study.

Out of 77 districts of Nepal, ST patients in our study were recorded from 33 different districts. More than 10 cases were reported from Dhading (39 cases), Nuwakot (16 cases), Kathmandu (16 cases), and Sarlahi (11 cases), each. The highest number of scrub typhus cases (108 cases) were reported from province number 3 (*Bagmati Pradesh*), followed by province number 2 (15 cases) and Gandaki province (11 cases) (Figure 3).

Among 154 ST patients, twenty (13%) responded that they had traveled to other districts within a month before the onset of the disease. Most of the patients (65%, n = 13) with travel history belonged to the age group 20–39 years. Higher cases of ST were recorded in patients with recent travel history compared to those without travel history. 

### 3.3. Different Febrile Illnesses and Mixed Seropositivity

Scrub typhus, dengue, leptospirosis, brucellosis, malaria, *kala-azar*, and typhoid were being tested as a single panel for febrile illness. Among the 2070 blood samples analyzed, 462 (22.3%) had at least one of the illnesses included in the panel (Table 3). Out of 9 malaria positive cases, 5 were positive for *Plasmodium vivax* and 4 for *P. falciparum*. Similarly, in the 52 brucellosis patients, 25 were positive for *Brucella abortus*, 22 were positive for *B. melitensis*, and 5 were positive for both *B. abortus* and *B. melitensis*. Two patients had mixed seropositivity of scrub typhus, dengue, and leptospirosis. The most common type of dual seropositivity was scrub typhus with dengue (n = 12), and scrub typhus with brucellosis (n = 9) (Table 3). 

### 3.4. Clinical Features of ST Patients 

Of 2070 febrile patients, 253 were seropositive for scrub typhus but details about symptomatology were accessible for 154 patients only. Since some of the blood samples collected were from patients clinically examined at different hospitals but visiting the STIDH for laboratory diagnosis, assessment of clinical symptomatology was not possible for those referred patients.

**Figure 4:**
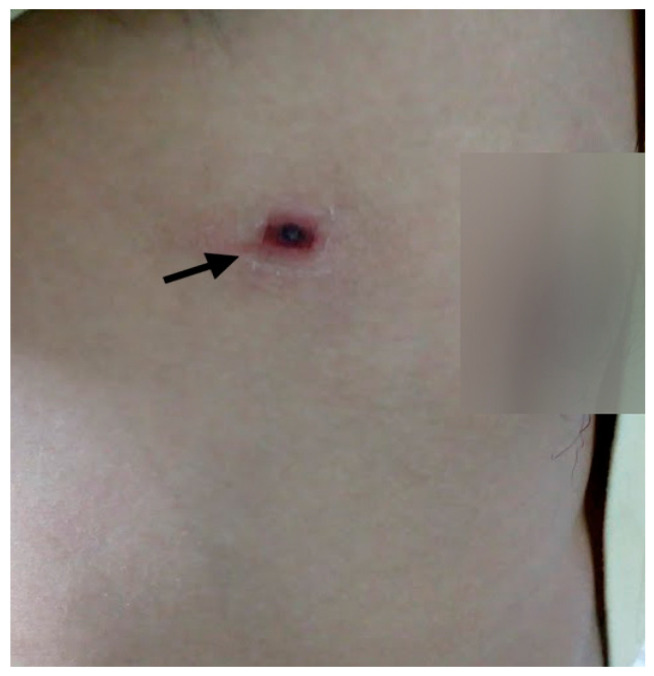
Pathognomonic eschar observed near the chest of a male ST patient.

Among the 154 ST patients, 61.7% (n = 95) had used at least one antibiotic before visiting the hospital (Supplementary Materials). Most of the patients (44.2%, n = 42) who used antibiotics prior to visiting the hospital were of age group 20–39 years (Table 2b). Out of 154 scrub typhus patients, apart from fever, the most common clinical symptoms were headache (79.2%), sweating (70.1%), breathing difficulty (51.3%), and redness of the eye (43.5%). The mean duration of fever was 9.34 days (Table 4). The duration of fever in the majority of ST patients (42.9 %, n = 66) was 5–9 days. No case with hepatomegaly and CNS involvement were recorded in scrub typhus patients during this study. Although shortness of breath was reported in some patients, neither of the ST patients was diagnosed with complications such as acute respiratory distress syndrome (ARDS). The pathognomonic eschar (Figure 4) was present only in 14 (9.1%) ST patients.

## 4. Discussion

Nepal has a diverse topography that includes Terai, hills, and mountains. Febrile illnesses including malaria, dengue, brucellosis, scrub typhus, and leptospirosis are more common in Terai regions with spreading towards the hilly regions [23,24]. The global decline of malaria revealed an array of acute undifferentiated febrile illnesses (UFI) which has taken a high toll on human health [25]. A large-scale study of UFI in tropical and subtropical regions has revealed that rickettsial diseases, predominantly ST and murine typhus, are among the leading causes of treatable UFI [26]. Among the included febrile patients in this study, the etiologies of only 22.3% of patients were identified. Since our study mainly focused on ST and other common febrile illnesses in Nepal, the febrile illness caused by many other etiologies may have been missed. 

As observed in this study, ST is the primary leading cause of treatable febrile illnesses based on seropositivity in Nepal. The prevalence of ST reported in our study (12.2%) is higher than the prevalence rate of 3.2% reported by a study based on another hospital in Kathmandu valley in 2001 [10]. The higher prevalence documented in the current study may be attributed to increased awareness among physicians about ST endemicity leading to an increased rate of scrub typhus case diagnosis, and easy availability of diagnostic tools (e.g., RDTs) compared to previous studies. Additionally, the seasonal variability of ST in Nepal might have contributed to the higher prevalence observed in our study. ST cases in Nepal peak from July to November which coincides with our study period [14,27]. In contrast to our findings, some studies have documented an even higher prevalence of ST in Nepal: 22% in 2004 [28] and 40.3% in 2015 [29]. Studies from Bangladesh, India, and Malaysia have reported ST prevalence rates of 23.7% in 2010 [30], 47.5% in 2008 [31], and up to 36% [32,33], respectively, which are higher than the rate observed in our study. These variations in the prevalence of ST may be because of different diagnostic tools/methods used. Since diagnostic tools/techniques may have varying specificity and sensitivity. Some researchers have tested both IgG and IgM [30] against ST, while others have tested either IgG only [32] or IgM only [29,30,31], this could be responsible for varying prevalence of ST. Furthermore, participant selection ‘bias’ such as febrile patients vs. febrile patients with clinical suspicion of scrub typhus could be another contributing factor to the observed variation in the prevalence of diseases. 

The prevalence of dengue (4.9%) reported in this study is lower than the prevalence range of 7–30% reported by other studies in Nepal in the past few years [28,34]. Similarly, our study found a relatively lower rate (2.9%) of typhoid fever among febrile patients compared to previous studies from Nepal (7.5–10.6%) [35,36] and Myanmar (47%) [37] which might be due to the Widal test used in our study to diagnose typhoid fever, which is known to have low sensitivity and specificity [38]. Increased access to safe drinking water and enhanced sanitation facilities among the Nepali population over the years or prior use of antibiotics might be other contributing factors as recorded with *Borrelia*, *Brucella*, and other infections [39,40]. Similarly, the seropositivity of leptospirosis and brucellosis observed in our study is much lower than the previous reports from different regions of Nepal that ranged from 4–21%, and 18%, respectively [10,28,41,42]. Although about 1% of the febrile cases in our study tested positive for visceral leishmaniasis, we were unable to distinguish whether the infection was an active or past infection. Despite having high sensitivity and specificity, the K39 strip test used in our study can remain positive long after the treatment for up to 3 years [43]. 

We observed mixed seropositivity of two or more different etiological agents in the same febrile patient. A similar observation of coinfection/mixed positivity has been made by other studies in Nepal (22.35%) [28] and India (4.1%) [44]. The apparently mixed infections or dual seropositivity of ST with other illnesses may have resulted due to the presence of residual IgM antibodies from past infections [28], or the dual infection may have resulted from actual concurrent infection by two or more agents. In addition, the dual seropositivity of scrub typhus and typhoid fever might be a result of the cross-reactive nature of the Widal test with scrub typhus [8]. Because the odds of serological cross-reactivity cannot be ruled out with RDTs, other approaches of confirmation such as PCR should be considered to reduce the possible misdiagnosis.

We observed more cases of ST in females compared to males, however, there was no significant difference in the ST cases between males and females. Females are disproportionately more confined to household and agricultural activities in Nepal which can increase the chances of exposure to *Orientia* vectors. A similar finding was reported from South Korea [45,46]. 

Most of the participants in our study responded that they had used at least one antibiotic prior to the hospital visit, and most ST patients were given antibiotics against typhoid fever. UFIs in Nepal are empirically treated as presumed enteric fever [47]. Since enteric fever is endemic to the country and similar nonspecific clinical presentations are observed in enteric fever and ST patients, it is apparent for a clinician or pharmacist to have recommended antibiotics against enteric fever. ST cases were relatively high in travelers than in patients without recent travel history. Travel to the village, recreational parks or shrub areas, and farming fields may increase the risk of intimate contact with rodents or vectors, thereby increasing the odds of being infected with *Orientia*. 

Along with fever; headache, sweating, breathing difficulty, and redness of the eye were the major clinical presentations among the included patients. All the enrolled patients had a complaint of fever with a mean febrile duration of about 9 days before attending the hospital. The reluctance of people to seek medical treatment unless the severity of symptoms increases, as well as the nonspecific nature of scrub typhus manifestations, causes febrile patients to often rely on self-medication to subside fever as an early response rather than seeking medical treatment early [48]. Breathing difficulty observed among ST patients may be associated with pulmonary involvement, which has been documented among ST patients in a previous study [2]. Ocular involvement in ST has also been documented previously [49,50]. In this study, 43.5% of ST patients presented with redness of the eye indicating possible ocular involvement. About one-tenth of total ST patients had presented with rashes and eschar. A varying prevalence of rashes (6–93%), and eschar (3–90%) has been reported in ST patients around the endemic region of the world [7,12,51,52,53,54,55,56,57,58,59,60]. The presence of characteristic eschar varies according to the geographical region [56,61,62] and the eschar-inducing capacity of *O. tsutsugamushi* strains [63]. Notably, indigenous people of endemic areas commonly have a less severe illness, often without any rash or eschar [64,65]. Although the presence of eschar could be specific, its painless nature and location on the body may not attract the attention of patients and clinicians. Eschar is mainly confined to the trunk, inguinal, genital, and axillary areas of the patients [52]. Due to the presence of eschar on such intimate parts of the body, patients may often hesitate to show the eschar to a clinician/physician, which might have also contributed to the lower prevalence of eschar in our study. As observed in this study, a low incidence of diarrhea was observed in a study reported from Sri Lanka (5%) in 2013 [52]. Unlike these findings, other studies have reported a higher prevalence of diarrhea in scrub typhus patients elsewhere [51,53,55]. Although recorded in very few ST patients, tinnitus may be associated with sensory neural hearing loss [66]. About half of the total ST patients in our study had thrombocytopenia while it was recorded in 46.9% of ST patients in Sri Lanka [52] and 25.7%–90% in India [53,54]. As we have observed in this study, an elevated level of liver enzymes such as ALP, SGPT, and SGOT is a well-documented manifestation of ST. In ST patients, elevated SGOT levels have been reported in 78–89%, elevated SGPT levels in 64%–92%, and elevated alkaline phosphatase (ALP) in 27–84% [49]. Although complications such as pneumonia, ARDS, renal failure, and meningoencephalitis are not uncommon in severe ST patients, the participants of this study did not report any such severe cases. This may be because either the patient enrolled were not at a severe stage of the infection or such complications may have been missed in the case of referred patients as they were referred just for the laboratory diagnosis and were not being treated in the STIDH.

This was a single hospital-based study conducted among patients of age ≥19 years, so the findings of this study might not necessarily represent the general population of Nepal. The specimens were not collected throughout the whole year. Thus, seasonal variation could not be presented. The use of rapid diagnostic test kits to identify the etiology of febrile patients might have caused an underestimation of the number of cases. Although the sensitivity and specificity of ImmuneMed scrub typhus rapid kit as claimed by the manufacturing company is 97.3% and 99.5%, researchers have documented it to be 87% and 94.64% for IgM, and 77.32% and 86.44% for IgG [67]. This low sensitivity and specificity might have either underestimated or overestimated the ST cases. All adult febrile patients attending STIDH were enrolled in this study; some of them were referred from some other hospital to STIDH. That referred patients collected the blood specimen in their respective hospitals and specimens were sent to STIDH for laboratory diagnosis, it is, therefore, blood/serum specimens for some referred patients were not available for tests other than serology. Hematological and biochemical data for some of those patients therefore could not be accessed.

## 5. Conclusions

This study shows a noticeable prevalence of scrub typhus infection in Nepal and therefore clinicians should consider ST as a major possible cause of acute febrile illness. Females and travelers are apparently at a greater likelihood of acquiring scrub typhus infection. Because scrub typhus and other febrile illness may present similar nonspecific presentations such as fever and headache, and pathognomonic eschar is less commonly seen in Nepalese patients, febrile patients must be carefully examined for scrub typhus in Nepal. To assist in the interpretation of serological results, future studies are required to define the background antibody prevalence and the persistence of residual antibodies from recent past infections.

## Figures and Tables

**Table 1:** List of RDT kits used for serodiagnosis of febrile illnesses.

Disease	Test Kit Used (S1%, S2%)	Manufacturer
Scrub typhus	ImmuneMed Scrub typhus Rapid (97.3%, 99.5%)	ImmuneMed Inc., Songpa-gu, Korea [19])
Dengue	ImmuneMed Dengue combo (NS1: 97.7%, 99.5%; IgM & IgG: 98.5%, 92.3%)	ImmuneMed Inc., Songpa-gu, Korea [19]
Leptospirosis	ImmuneMed leptospira Rapid (96.4%, 98.4%)	ImmuneMed Inc., Songpa-gu, Korea [19]
Brucellosis	Brucel^®^ antigen solution ‘A’ and solution ‘B’ (70%, 70%)	Tulip Diagnostics, Goa, India [20]
Kala-azar	Kalazar Detect^TM^ (>90%, >90%)	InBios International Inc., Songpa-gu, USA [21]
Enteric fever	TYDAL^®^ (Widal test) (70%, 70%)	Tulip Diagnostics, Goa, India [22]

S1: sensitivity, S2: specificity as mentioned in the product catalog/manufacturer’s website.

**Table 2:** Demographic distribution, history of antibiotics usage, and laboratory investigation of patients. (**A**)Distribution of total febrile patients based on age-group (n = 2070); (**B**) Distribution of ST patients using antibiotics before the hospital visit (n = 95); (**C**) Biochemical and hematological findings of ST patients (n = 139).

**A: Distribution of Total Febrile Patients Based on Age-Group (n = 2070)**			
**Age Category**	**Frequency**		**Total**
	**Male N (%)**	**Female N (%)**	
<20	196 (16.5)	138 (15.7)	334 (16.14)
20–39	493 (41.4)	359 (40.8)	852 (41.15)
40–59	347 (29.1)	266 (30.3)	613 (29.61)
60–79	136 (11.4)	108 (12.3)	244 (11.79)
80 or above	19 (1.6)	8 (0.9)	27 (1.31)
Total	1191 (57.5)	879 (42.5)	2070 (100)
**B: Distribution of ST Patients Using Antibiotics before the Hospital Visit (n = 95)**			
Age group		Frequencies, n (%)	
Less than 20		15 (15.8)	
20–39		42 (44.2)	
40–59		30 (31.6)	
60–79		6 (6.3)	
80 and above		2 (2.1)	
Total		95 (100)	
**C: Biochemical and Hematological Findings of ST Patients (n = 139)**			
Parameters *		Frequencies, n (%)	
WBC count			
Low		11 (7.9)	
High		3 (2.2)	
Thrombocytopenia		77 (55.4)	
Raised ALP		44 (31.7)	
Raised SGPT		53 (38.1)	
Raised SGOT		85 (61.2)	
Raised BT		9 (6.5)	
Raised BD		13 (9.4)	
Raised serum creatinine		14 (10.1)	

WBC: White blood corpuscles, SGPT: Serum Glutamic Pyruvic Transaminase, SGOT: Serum Glutamic Oxaloacetic Transaminase, BT: Total bilirubin, BD: Direct bilirubin, ALP: Alkaline phosphatase, * see Supplementary Materials for reference range.

**Table 3:** Mixed seropositivity in febrile patients.

Febrile Illness(es)	Frequency (% of Total Febrile Patients)
ST ^+^	221 (10.7)
DEN ^+^	81 (3.9)
LEP ^+^	3 (0.1)
BRU ^+^	39 (1.9)
MAL ^+^	7 (0.3)
KAL ^+^	18 (0.9)
TYP ^+^	52 (2.5)
ST ^+^ DEN ^+^	12 (0.6)
ST ^+^ LEP ^+^	2 (0.1)
ST ^+^ BRU ^+^	9 (0.4)
ST ^+^ MAL ^+^	1 (0.0*)
ST ^+^ KAL ^+^	1 (0.0*)
ST ^+^ TYP ^+^	5 (0.2)
DEN ^+^ LEP ^+^	2 (0.1)
DEN ^+^ BRU ^+^	1 (0.0*)
DEN ^+^ MAL ^+^	1 (0.0*)
DEN ^+^ TYP ^+^	2 (0.1)
BRU ^+^ KAL ^+^	1 (0.0*)
BRU ^+^ TYP ^+^	2 (0.1)
ST ^+^ DEN ^+^ LEP ^+^	2 (0.1)
Total	462 (22.3)

**Key:**^+^ Seropositive, DEN: Dengue, LEP: Leptospirosis, ST: Scrub typhus, BRU: Brucellosis, MAL: Malaria, KAL: Kala-azar, TYP: typhoid fever. *(0.0 means mixed seropositivity in 1 febrile cases among 2070 patients).

**Table 4:** Clinical features of ST patients.

Presented Signs and Symptoms	Value	*p*-Value (χ^2^)
Fever, n (%)	154 (100)	
Duration of fever before hospital visit (days), mean ± SD (Range)	9.34 ± 4.84 days (1–30)	
Headache, n (%)	122 (79.2)	<0.01
Sweating, n (%)	108 (70.1)	<0.01
Difficulty in breathing, n (%)	79 (51.3)	<0.01
Redness of eyes, n (%)	67 (43.5)	<0.01
Rashes, n (%)	15 (9.7)	
Eschar, n (%)	14 (9.1)	
Diarrhea, n (%)	5 (3.2)	
Tinnitus, n (%)	3 (1.9)	
Lymphadenopathy, n (%)	3 (1.9)	
Splenomegaly, n (%)	1 (0.6)	
Hepatomegaly, n (%)	0 (0.0)	

## Data Availability

All relevant data are presented in the main text as tables or figures. Data can be made available upon reasonable request to the corresponding author.

## Supplementary Materials

The following are available online at https://www.mdpi.com/article/10.3390/tropicalmed6020078/s1, Questionnaire, Table S1: The reference range for biochemical and hematological parameters, Table S2: List of antibiotics used by patients prior to the hospital visit, Table S3: A list of traveled places by ST patients are included.

## Abbreviations

ALP	Alkaline phosphatase
BD	Direct bilirubin
BT	Total bilirubin
LMIC	Low-and middle-income country
PCR	Polymerase chain reaction
RDT	Rapid diagnostic test
SGOT	Serum glutamic oxaloacetic transaminase
SGPT	Serum glutamic pyruvic transaminase
TLC	Total leukocyte count
UFI	Undifferentiated febrile illness
WBC	White blood corpuscle
