# Supplementary material for: Seroprevalence and Clinical Features of Scrub Typhus among Febrile Patients Attending a Referral Hospital in Kathmandu, Nepal
Source: Trop Med Infect Dis. 2021 May 13;6(2):78. doi: 10.3390/tropicalmed6020078 (PMC8163188; doi:10.3390/tropicalmed6020078)
Supplement: Supplementary file 1 [file tropicalmed-06-00078-s001.zip › tropicalmed-1162515-supplementary.pdf]

Tropical Medicine and Infectious Disease

MDPI

Supplementary

Seroprevalence and Clinical Features of Scrub Typhus among Febrile Patients Attending a Referral Hospital in Kathmandu, Nepal

Anil Pokhrel [1], Binod Rayamajhee [2], Saroj Khadka [1], Sandeep Thapa [3], Samjhana Kapali [1], Sher Bahadur Pun [4], Megha Raj Banjara [1], Prakash Joshi [5], Binod Lekhak[1] and Komal Raj Rijal [1,*]

Questionnaire

" Seroprevalence and Clinical Features of Scrub Typhus in Febrile Patients Attending Sukraraj Tropical and Infectious Disease Hospital"

Questionnaire

Date ....

Name of patient ....

Lab ID ....

Age ....

Gender

Male ☐ Female ☐

Address ....

Patient Type:

OPD ☐ Inpatient ☐

Prior Treatment with Antibiotics:

Yes ☐ No ☐

If Yes, Name of Antibiotics and Duration of Dose ....

Travel History

Yes ☐ No ☐

If Yes, Name of travelled place ....

Symptomatology:

Fever, If present, Days of fever ....
Headache
Eschar
Rash
Sweating

Tropical Medicine and Infectious Disease

MDPI

Redness in the Eye
Breathing Difficulty
Diarrhoea
Tinnitus
Lymphadenopathy
Hepatomegaly
Splenomegaly
Complication, if present, mention…………….

Table S1. Reference range for biochemical and hematological parameters.

Test	Reference Range *	Unit
TLC	4000-11000	cells/cumm
Platelet	150-400	×1000/cumm
Bilirubin Total	0.3-0.4	mg/dl
Bilirubin Direct	0.0-0.4	mg%
Alkaline phosphatase	M: 53-128, F: 42-98, C: 54-369	U/L
SGPT	Upto 42	U/L
SGOT	Upto 37	U/L
Creatinine	0.4-1.4	mg%

*As defined by Sukraraj tropical and infectious disease hospital, TLC: Total Leucocyte Count; cumm: cubicmilimeter; mg: milligram; dl: decilitre; L: Litre; SGPT: Serum Glutamic Pyruvic Transaminase; SGOT: Serum glutamic oxaloacetic transaminase; M: male; F: Female; C: children

Table S2. List of travelled places by ST patients.

Name of Place/District Travelled	Number of Patients (% of total scrub typhus patients)
Chitwan	4 (2.6)
Gulmi	2 (1.3)
Sindhupalchock	2 (1.3)
Bardiya	1 (0.6)
Dhading	1 (0.6)
Dhangadi	1 (0.6)
Gorkha	1 (0.6)
Makawanpur	1 (0.6)
Kabhre	1 (0.6)
Rautahat	1 (0.6)
Sarlahi	1 (0.6)
Sindhuli	1 (0.6)
Udayapur	1 (0.6)
Forest exposure	1 (0.6)
Recently returned from India	1 (0.6)

Tropical Medicine and Infectious Disease

MDPI

Table S3: List of antibiotics used by ST patients prior to hospital visit

Name of Antibiotics	Number of Patients (%)
Against typhoid	12 (12.6)
Cefixime	5 (5.1)
Against UTI	2 (2.1)
Azithromycin	2 (2.1)
Ceftriaxone	1 (1.1)
Cefpodoxime	1 (1.1)
Cloxacillin	1 (1.1)
Meprazole	1 (1.1)
Norfloxacin	1 (1.1)
Montaz (Ceftriaxone + Tazobactam)	1 (1.1)
Used but unable to identify neither antibiotic nor disease	68 (71.5)

UTI: Urinary tract infection
